# Phytochemical composition and bioactivities of *Satureja montana* L. and *Satureja hortensis* L.: Culinary herbs with antidiabetic, anticholinesterase, and antioxidant potential

**DOI:** 10.1371/journal.pone.0332178

**Published:** 2025-09-15

**Authors:** Furkan Coban, Yuzhou Lan, Gül Yetisgin, Hafize Yuca, Bilge Aydın, Hilal Angın, Betül Demirci, Songül Karakaya

**Affiliations:** 1 Department of Field Crops, Faculty of Agriculture, Atatürk University, Erzurum, Türkiye; 2 Department of Plant Breeding, The Swedish University of Agricultural Sciences, Lomma, Sweden; 3 Department of Pharmacognosy, Faculty of Pharmacy, Atatürk University, Erzurum, Türkiye; 4 Department of Pharmacognosy, Faculty of Pharmacy, Erzincan Binali Yıldırım University, Erzincan, Türkiye; 5 Department of Plant Production and Technologies, Faculty of Applied Sciences, Muş Alparslan University, Muş, Türkiye; 6 Department of Pharmacognosy, Faculty of Pharmacy, Anadolu University, Eskişehir, Türkiye; 7 Department of Pharmaceutical Botany, Faculty of Pharmacy, Atatürk University, Erzurum, Türkiye; Universidad Autonoma de Chihuahua, MEXICO

## Abstract

The increasing global burden of oxidative stress-related conditions, diabetes, and neurodegenerative disorders underscores the urgent need for multi-targeted, plant-based therapeutic agents. In this context, the present study aimed to evaluate the antidiabetic, antioxidant, and neuroprotective properties of various *Satureja hortensis* and *Satureja montana* extracts and essential oils. In vitro biological activities were assessed through α-glucosidase, acetylcholinesterase (AChE), and butyrylcholinesterase (BChE) inhibition assays, along with DPPH• and ABTS• ⁺ radical scavenging assays. At a concentration of 100 µg/mL, *S. hortensis* root methanol extract (ShRME) and herb water extract (ShHWE) exhibited strong α-glucosidase inhibitory activity (69.88% and 71.23%, respectively). Conversely, *S. montana* herb water extract (SmHWE) and methanol extract (SmHME) showed the highest DPPH• radical scavenging activity (36.28% and 24.37%, respectively*). S. montana* essential oil (SmEO) demonstrated notable inhibition of AChE (32.58%) and BChE (41.62%) at 1000 µg/mL, while *S. hortensis* essential oil (ShEO) inhibited BChE by 44.39%. GC-MS analysis revealed that SmEO primarily contained carvacrol (43.72%), γ-terpinene (17.24%), and p-cymene (14.56%), while ShEO was rich in thymol (39.84%), γ-terpinene (20.16%), and p-cymene (13.72%). These phenolic monoterpenes are likely contributors to the observed antioxidant and cholinesterase inhibitory activities. Principal component analysis explained 74.8% of the total variance and clearly separated the samples based on their activity profiles. Extracts were closely associated with glucose-regulating effects, while essential oils clustered with enzyme-inhibiting neuroprotective activities. These findings highlight the multifunctional therapeutic potential of *Satureja* species and support their further investigation as candidates for the development of plant-derived agents against oxidative stress, diabetes, and neurodegenerative diseases.

## Introduction

Diabetes mellitus (DM) is a metabolic disorder with hyperglycemia and complications like retinopathy, nephropathy, neuropathy, and cardiovascular disease [1]. Its impact on cognitive function is unclear. Type 1 DM involves insulin deficiency, with cognitive dysfunction affected by onset age, glycemic control, and duration. Type 2 DM, the most common form, accounts for 90–95% of cases, marked by insulin resistance and linked to obesity, hypertension, and early cognitive impairments [2].

Dementia, particularly Alzheimer’s disease (AD), is common in older adults and has a significant societal impact. Despite research, no cure exists for AD, which is characterized by amyloid-β plaques and tau tangles. The mechanisms behind their accumulation are unclear, and AD’s complexity suggests it is a syndrome requiring a multifaceted treatment approach [3].

The International Diabetes Federation reports 589 million people with DM, with 252 million undiagnosed adults in the latest edition of IDF Diabetes Atlas (2025). Furthermore, by 2050, the total number of people diagnosed with DM is expected to rise to 853 million. DM has caused 6.7 million deaths and imposes a heavy financial burden, especially on the poor. AD and other dementias affect over 55 million people globally, with a new case every 3 seconds. Both DM and AD heavily impact global health, particularly women, who represent two-thirds of AD patients and face a 30% higher mortality rate. These figures highlight the need for ongoing research and progress [4].

DM is an independent risk factor for dementia, particularly AD and vascular dementia (VaD). Proposed mechanisms include vascular issues, hyperglycemia, insulin resistance, and impaired β-amyloid degradation, but these remain unproven. Type 2 diabetes is linked to faster cognitive decline, and diabetic AD patients have higher mortality rates. The connection between AD and diabetes is often called type 3 diabetes mellitus (T3DM) [5,6].

Oxidative stress, driven by excess reactive oxygen and nitrogen species, increases in DM due to hyperglycemia, leading to protein glycation, lipid peroxidation, and cellular dysfunction. While the exact cause of AD is unknown, oxidative stress is widely recognized as a key factor in its development [7,8].

Natural compounds from plants, animals, and microorganisms are valuable for drug discovery. DM and prediabetes heighten the risk of neurodegenerative diseases like vascular dementia and AD, making diabetic control crucial. Despite this, several natural compounds have been reported to reduce vascular damage, neuroinflammation, and neurodegeneration, offering neuroprotective benefits [9].

*Satureja hortensis* L. (summer savory) is an annual aromatic herb of the *Lamiaceae* family, native to southern Europe and the Mediterranean, and now cultivated worldwide, including Türkiye, where it is locally known as ‘Koc Out’. Its essential oil, rich in carvacrol, thymol, and other terpenoids, is valued for its antimicrobial, antidiarrheal, and antioxidant properties. Traditionally, the plant has been used as a remedy for cramps, muscle pain, nausea, indigestion, diarrhea, and infections. The leaves, flowers, and stems are commonly used as tea or in spice mixtures, adding aroma and flavor to foods, while its medicinal applications include antispasmodic and sedative effects [10–12].

*S. montana* L., or winter savory, is an evergreen perennial shrub from the Lamiaceae family, native to Southern Europe and commonly found in rocky, sunny Mediterranean regions. It grows to 20–30 cm in height, with woody, branched stems and lanceolate, glossy dark-green leaves. The plant produces white to pale pink-violet flowers with purple-spotted tips, blooming between July and September, and forms fruits of four nutlets. Grown in different habitats, this species exhibits significant morphological variability, including differences in leaf shape and pubescence, as well as floral and fruit characteristics. Found in regions such as northeastern Portugal and Madeira, *S. montana* thrives in arid and rocky environments but presents taxonomic challenges due to its polymorphic nature [13–15].

Although *Satureja hortensis* (summer savory) has long been employed in traditional medicine for its carminative, antispasmodic, diuretic, antiseptic, and analgesic properties particularly in the treatment of digestive disorders, muscle pain, and infections there is no ethnobotanical evidence supporting its use in managing diabetes or neurodegenerative diseases such as Alzheimer’s disease. In contrast, contemporary phytochemical and pharmacological studies have revealed that *S. hortensis* contains a wide array of bioactive compounds, including phenolic acids (e.g., rosmarinic acid, caffeic acid, quercetin) and monoterpenoids (e.g., carvacrol, thymol), which have demonstrated potent antioxidant, anti-inflammatory, anticholinesterase, and antidiabetic effects in both in vitro and in vivo models. For instance, methanolic extracts of *S. hortensis* have shown neuroprotective potential by reducing oxidative stress and apoptotic markers in the hippocampus, while other *Satureja* species have been associated with glucose- and lipid-lowering effects in diabetic animal models [16–19]. This discrepancy between traditional usage and experimental evidence highlights a relevant scientific gap and reinforces the rationale for investigating *S. hortensis* as a potential source of antidiabetic and neuroprotective agents.

In a similar context, *Satureja montana* (winter savory), although primarily recognized as a culinary and aromatic herb in Mediterranean cuisine, has also been traditionally used for its digestive, antiseptic, and antispasmodic properties. However, like *S. hortensis*, no ethnobotanical evidence supports its use in the treatment of diabetes or neurodegenerative diseases [20]. Recent phytochemical investigations have demonstrated that *S. montana* is rich in phenolic acids (e.g., rosmarinic acid, caffeic acid) and monoterpenoids (e.g., carvacrol, thymol, p-thymol), which exhibit potent antioxidant, anti-inflammatory, and enzyme-inhibitory activities [21]. Notably, its essential oil has been shown to significantly inhibit acetylcholinesterase (AChE), an enzyme closely associated with neurodegenerative processes, indicating potential neuroprotective properties [20]. Furthermore, the synergistic action of carvacrol, thymol, and thymoquinone in its volatile extracts enhances antioxidant capacity, which is critical in mitigating oxidative stress implicated in both diabetes and neurodegeneration [22,23].

Despite the lack of traditional use for metabolic or neurological conditions, the promising pharmacological profile of *S. montana* provides a strong rationale for its inclusion in the current study. Accordingly, we aimed to evaluate its potential antidiabetic activity via α-glucosidase and α-amylase inhibition, as well as its neuroprotective effects through AChE inhibition, antioxidant capacity, and modulation of neurodegeneration-related biomarkers.

This research investigates the inhibitory effects of essential oils, methanol, and water extracts derived from the aerial parts with flowers of *S. hortensis* and *S. montana* on acetylcholinesterase and butyrylcholinesterase enzymes. Their potential to inhibit α-amylase and α-glucosidase enzymes was also assessed, along with an evaluation of their antioxidant activities. Additionally, the chemical compositions of the essential oils were determined by GC-MS/MS analysis.

## Materials and methods

### Plant materials

Two *Satureja* species were used in this study: a local population of *Satureja hortensis* L. and *Satureja montana* L. The seeds of *S. hortensis* were obtained from Ethno-Garden (Poland) and cultivated under controlled greenhouse conditions at the Faculty of Agriculture, Atatürk University (Erzurum, Türkiye). The plants were grown in plastic pots (20 cm diameter) containing a peat-perlite mixture (2:1, v/v). Greenhouse conditions were maintained at day/night temperatures of 24 ± 2 °C/ 18 ± 2 °C, relative humidity of 60–70%, and natural photoperiod during the spring season. Irrigation was carried out regularly to maintain consistent soil moisture, and no chemical fertilizers or pesticides were applied. Aerial parts were harvested during the full flowering stage. The aerial parts (herba) of *Satureja montana* were provided in fresh form by the Department of Vegetable and Medicinal Plants, Warsaw University of Life Sciences (WULS-SGGW), Poland. The plant material, consisting of flowering shoots, was collected during the blooming period and shipped under appropriate conditions to preserve phytochemical integrity. Upon arrival, the material was processed and dried under standardized laboratory conditions and subsequently used as authenticated botanical raw material. The taxonomic identification of both species was verified. The seed material of *Satureja hortensis* was registered under the accession number ZFTB00SS05, while the herbarium specimen of *Satureja montana* (herba) was deposited in the Herbarium of the Department of Field Crops, Atatürk University, under the herbarium number ZFTB00SH03.

### Extraction

In this study, the dried aerial parts with flowers of *Satureja hortensis* (30 g) and *Satureja montana* (30 g) were cut into small pieces and subjected to methanolic extraction via maceration at room temperature for 3 days and 8 hours. The resulting extracts were filtered and concentrated under vacuum to dryness, yielding 5.12 g (17.07% w/w) and 4.91 g (16.37% w/w), respectively.

For the decoction preparation, 30 g of aerial parts with flowers of *S. hortensis* and *S. montana* were weighed and ground into a fine powder. Distilled water (150 mL) was added to the powder, and the mixture was macerated. After filtration, the extract was frozen at −80°C and then lyophilized to obtain a dry powder. The final extract yields were 6.09 g (20.30% w/w) for S. hortensis and 7.45 g (24.83% w/w) for *S. montana.*

For essential oil extraction, 200 g of each plant material was subjected to hydrodistillation using a Clevenger-type apparatus for 3 hours. The extracted oils were dried over anhydrous sodium sulfate and stored at +4°C in the dark until further analysis and testing. The essential oil of *S. hortensis* was analyzed using GC and GC-MS techniques, following the methodology outlined by Karakaya et al. [24]. The essential oil of *S. montana* was analyzed using GC and GC-MS techniques, as described below.

### GC-FID analysis

The GC analysis was carried out using an Agilent 7890B GC System. FID detector temperature was 250°C. Relative percentage amounts of the separated compounds were calculated from FID chromatograms.

### GC-MS analysis

The GC-MS analysis was carried out with an Agilent 7890B GC 5977B Mass Selective Detector System. Innowax FSC column (60 m x 0.25 mm, 0.25 μm film thickness) was used with helium as carrier gas (0.7 mL/min). GC oven temperature was kept at 60°C for 10 min and programmed to 220°C at a rate of 4°C/min, and kept constant at 220°C for 10 min and then programmed to 240°C at a rate of 1°C/min., total 80 min. Split ratio was adjusted at 40:1. The injector temperature and ion source temperature were set at 250°C and 230°C, respectively. Mass spectra were recorded at 70 eV. Mass range was from m/z 35–450.

### Identification of components

Identification of the essential oil components was carried out by comparing their relative retention times with those of authentic samples or by comparison of their relative retention index (RRI) to a series of n-alkanes. Computer matching against commercial (Wiley 9-Nist 11 Mass Spectral Database, and in-house “Başer Library of Essential Oil Constituents” built up by genuine compounds and components of known oils was used for the identification.

### DPPH radical scavenging activity assay

The antioxidant capacity was evaluated through their ability to scavenge DPPH^•^ free radicals, using the method originally described by Blois [25] with minor modifications introduced by Aydın et al. [26]. In this assay, *α*-tocopherol and trolox were employed as reference antioxidants. A 1 mM DPPH^•^ solution in ethanol served as the radical source. Initially, the inhibition percentages of the standards at various concentrations (1–100 μg/mL) were measured to determine an appropriate concentration range for the test samples. Based on these values, calibration curves were generatedwith correlation coefficients (r) at 0.99, indicating strong linearity. Subsequently, the extracts were tested in the 10–100 μg/mL concentration range through serial dilutions. Each sample was mixed with the DPPH^•^ solution, and the reaction mixtures were incubated in the dark. Absorbance was measured at 517 nm against a blank composed of 99% ethanol. All experiments were conducted in triplicate to ensure reliability. The radical scavenging activity of the extracts was expressed as a percentage of inhibition, calculated using the following equation: % Inhibition = [(A_control_ -A_Sample_)/ A_control_] x 100. Data for this assay is presented in S1 Table.

### ABTS radical scavenging activity assay

The antioxidant potential of *Satureja* extracts was assessed through their ABTS^•⁺^ radical scavenging activity, following the procedure originally described by Re et al. [27], with minor modifications introduced by Aydın et al. [26]. In this method, *α*-tocopherol and trolox were employed as reference antioxidants, and their activity was tested using a 2 mM ABTS• ⁺ solution. The inhibition percentages of the standards at defined concentrations (1–100 μg/mL) were analyzed to establish an effective concentration range for evaluating the plant extracts. Calibration curves generated from these values yielded correlation coefficients (r) of 0.99, indicating high linearity and reliability. Based on the determined range, serial dilutions of the extracts (10–100 μg/mL) were prepared and reacted with the ABTS^•⁺^ solution. After incubation, the reduction in absorbance was measured at 734 nm against a blank solution containing phosphate buffer in triplicate. The radical scavenging ability of the extracts was expressed as percentage inhibition and calculated using the following equation: % Inhibition = [(A_control_ -A_Sample_)/ A_control_] x 100. Data for this analysis is presented in S2 Table.

### α-Glucosidase inhibitory activity assay

The inhibitory activity against α-glucosidase was evaluated using a modified protocol based on the method reported by Bachhawat et al. [28]. In this assay, 50 µL of phosphate buffer (50 mM, pH 6.9), 10 µL of α-glucosidase enzyme solution (1 U/mL), and 20 µL of plant extract at varying concentrations (1–5000 µg/mL) were added to each well of a 96-well microplate. The mixture was pre-incubated at 37 °C for 5 minutes. Following this, 20 µL of the substrate solution, p-nitrophenyl-α-D-glucopyranoside (pNPG, 3 mM), was added to initiate the reaction, and the plate was further incubated at 37 °C for 30 minutes. The enzymatic reaction was then stopped by the addition of 50 µL of 0.1 M sodium carbonate. All reagents and solutions were prepared using the phosphate buffer. Acarbose served as the reference standard. The release of p-nitrophenol (pNP), which indicates enzyme activity, was quantified by measuring the absorbance at 405 nm. All experiments were conducted in triplicate, and the percentage inhibition of α-glucosidase was calculated using the formula below: Inhibition (%) = (1 - ΔA405sample/ ΔA405control) x 100. The data for this assay is presented in S3 Table.

### α-Amylase inhibitory activity assay

The α-amylase inhibitory potential of the samples was assessed using a modified version of the procedure described by Nampoothiri et al. [29]. Briefly, 100 µL of the test sample (ranging from 1 to 5000 µg/mL) was mixed with 100 µL of 1% soluble starch solution prepared in 20 mM sodium phosphate buffer (pH 6.9) containing 6 mM sodium chloride. The mixtures were pre-incubated at 25 °C for 10 minutes in a 96-well microplate. Subsequently, 100 µL of porcine pancreatic α-amylase (0.5 mg/mL) was added to initiate the reaction, and the plate was incubated for an additional 10 minutes at the same temperature. The enzymatic reaction was halted by adding 200 µL of dinitrosalicylic acid (DNS) reagent to each well, followed by heating at 100 °C for 5 minutes. After cooling the samples to room temperature, 50 µL of each reaction mixture was transferred into a new 96-well microplate, and 200 µL of distilled water was added to each well to dilute the contents. The absorbance was then recorded at 540 nm using a microplate reader. Acarbose was employed as the standard inhibitor for comparison. The percentage inhibition of α-amylase activity was calculated using the following equation: Inhibition (%) = (1 - ΔA405sample/ ΔA405control) x 100. The data for this analysis is presented in S4 Table.

### Acetylcholinesterase (AChE) and Butyrylcholinesterase (BChE) inhibitory activities

The inhibitory effects of the samples on acetylcholinesterase (AChE) and butyrylcholinesterase (BChE) enzymes were assessed using a modified version of the method proposed by Ingkaninan et al. [30]. The assays were conducted in a 96-well microplate format. For each reaction, 125 µL of Ellman’s reagent (3 mM 5,5’-dithiobis-(2-nitrobenzoic acid), DTNB), 25 µL of substrate solution (15 mM acetylthiocholine iodide for AChE or butyrylthiocholine iodide for BChE), 50 µL of Tris-HCl buffer (50 mM, pH 8.0), and 25 µL of the test sample were added to the wells. The reaction was initiated by the addition of 25 µL of the corresponding enzyme solution. The mixtures were then incubated at room temperature for 10 minutes for AChE and 15 minutes for BChE. Following incubation, the absorbance was measured at 405 nm to determine the formation of the yellow-colored 5-thio-2-nitrobenzoate anion, which correlates with enzyme activity. Donepezil was used as the standard reference inhibitor. All experiments were performed in triplicate, and the percentage inhibition was calculated to evaluate the cholinesterase inhibitory potential of the samples. The percentage of enzyme inhibition was calculated using the following formula: Inhibition (%) = (1 - ΔA405sample/ ΔA405control) x 100. The data for AChE and BChE inhibitory activities are presented in S5 Table and S6 Table.

### Data analysis

All statistical analyses and visualizations were performed using R software (RStudio 2024.12.0). Data wrangling and visualization were carried out using the tidyverse and ggplot2 packages, while group differences were assessed through one-way ANOVA followed by Duncan’s multiple range test using the agricolae package. Pairwise relationships were examined via correlation analyses (GGally, ggpubr), and the distribution of samples based on biological activity was evaluated using principal component analysis (PCA) with the FactoMineR and factoextra packages. Data points marked as nd (“not determined”) were excluded from the statistical analyses.

## Results

### Essential oil extraction and analysis

**Fig 1:**
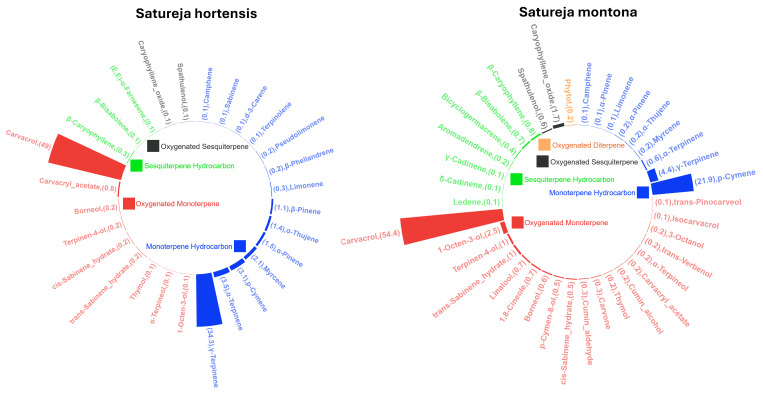
Radial treemaps of the chemical constituents of a) *Satureja hortensis* (ShEO) and b) *Satureja montana* (SmEO) essential oils analyzed by GC–MS. Each treemap illustrates the compound names and their corresponding relative peak areas (%). Different colors represent different categories of compounds.

The essential oils extracted from the flowering aerial parts of *Satureja hortensis* and *Satureja montana* yielded 0.20% (v/w) and 0.16% (v/w), respectively. Visually, the essential oil of *S. hortensis* appeared light yellow, while *S. montana* oil was distinctly orange, indicating compositional differences. A total of 40 compounds were identified in the essential oil of *S. hortensis*, representing 99.7% of the total oil content, with carvacrol (49.0%) and γ-terpinene (34.3%) being the most abundant. In the essential oil of *S. montana*, 46 compounds were characterized, accounting for 96.6% of the oil. Oxygenated monoterpenes constituted the major compound group in both oils comprising 50.8% in *S. hortensis* and 60.5% in *S. montana* followed by monoterpene hydrocarbons. The full list of identified compounds, their retention indices, and relative percentages are provided in **Table 1**. Compound classification by chemical group is summarized in **Fig 1**, which highlights the relative distribution of major chemical classes within each essential oil.

**Table 1:** Chemical composition of essential oils from *Satureja hortensis* and *Satureja montana.*

RRI	Compound	*S. hortensis* (%)	*S. montana (*%)	IM
1014	Tricyclene	tr	–	MS
1032	*α*-Pinene	1.5	0.2	RRI, MS
1035	*α*-Thujene	1.4	0.2	MS
1076	Camphene	0.1	0.1	RRI, MS
1118	*β*-Pinene	1.1	0.1	RRI, MS
1132	Sabinene	0.1	tr	RRI, MS
1159	*δ*-3-Carene	0.1	–	MS
1174	Myrcene	2.1	0.2	RRI, MS
1176	α-Phellandrene	–	tr	RRI, MS
1183	*Pseudolimonene*	0.2	–	MS
1188	*α*-Terpinene	3.5	0.6	RRI, MS
1203	Limonene	0.3	0.1	RRI, MS
1213	1,8-Cineole	–	0.7	RRI, MS
1218	*β*-Phellandrene	0.2	–	RRI, MS
1225	(*Z*)-3-Hexenal	tr	–	MS
1255	*γ*-Terpinene	34.3	4.4	RRI, MS
1280	*p*-Cymene	3.1	21.9	RRI, MS
1290	Terpinolene	0.1	tr	RRI, MS
1393	3-Octanol	tr	0.2	RRI, MS
1452	*α*-*p*-Dimethyl styrene	tr	–	MS
1452	1-Octen-3-ol	0.1	2.5	MS
1474	*trans*-Sabinene hydrate	0.2	1.0	MS
1479	(*E,Z*)-2,4-Heptadienal	tr	–	MS
1497	α-Copaene	–	tr	RRI, MS
1507	(*E,E*)-2,4-Heptadienal	tr	–	MS
1528	α-Bourbonene	–	tr	MS
1553	Linalool	–	0.7	RRI, MS
1556	*cis*-Sabinene hydrate	0.2	0.5	MS
1611	Terpinen-4-ol	0.2	1.0	RRI, MS
1612	*β*-Caryophyllene	0.3	0.8	RRI, MS
1628	Aromadendrene	–	0.2	MS
1630	4-Terpinenyl acetate	tr	–	MS
1670	trans-Pinocarveol	–	0.1	RRI, MS
1683	trans-Verbenol	–	0.2	MS
1706	*α*-Terpineol	0.1	0.2	RRI, MS
1708	Ledene	–	0.1	MS
1719	Borneol	0.2	0.6	RRI, MS
1741	*β*-Bisabolene	0.1	0.7	RRI, MS
1751	Carvone	tr	0.3	RRI, MS
1755	Bicyclogermacrene	tr	0.4	MS
1758	(*E,E*)-*α-*Farnesene	0.1	–	MS
1773	δ-Cadinene	–	0.1	MS
1776	γ-Cadinene	–	0.1	MS
1784	*(E)*-*α-*Bisabolene	tr	–	MS
1802	Cumin aldehyde	–	0.3	RRI, MS
1804	Myrtenol	–	tr	MS
1864	p-Cymen-8-ol	–	0.5	RRI, MS
1867	Thymyl acetate	–	tr	RRI, MS
1890	Carvacryl acetate	0.8	0.2	RRI, MS
2008	Caryophyllene oxide	0.1	1.7	RRI, MS
2113	Cumin alcohol	–	0.2	RRI, MS
2144	Spathulenol	0.1	0.6	RRI, MS
2181	Isothymol	tr	tr	MS
2198	Thymol	0.1	0.2	RRI, MS
2221	Isocarvacrol	tr	0.1	MS
2239	Carvacrol	49.0	54.4	RRI, MS
2622	Phytol	–	0.2	MS
	Monoterpene Hydrocarbons	48.1	28.5	
	Oxygenated Monoterpenes	50.8	60.5	
	Sesquiterpene Hydrocarbons	0.4	2.4	
	Oxygenated Sesquiterpenes	0.2	2.3	
	Diterpenes	**–**	0.2	
	Others	0.2	2.7	
	Total	**99.7**	**96.6**	

(RRI: Relative retention indices calculated against n-alkanes; %: calculated from FID data; tr: Trace (< 0.1%), IM: Identification method based on the relative retention indices (RRI) of authentic compounds on the HP Innowax column; MS, identified on the basis of computer matching of the mass spectra with those of the Wiley and MassFinder libraries and comparison with literature data.

### Antioxidant activity

To comprehensively evaluate the antioxidant potential of the tested samples, both ABTS•⁺ and DPPH• spectrophotometric assays were employed. The experimental results are presented in **Table 2**. In the radical scavenging efficacy evaluation, the reference compound Trolox demonstrated the highest inhibition at 70 µg/mL in the ABTS• ⁺ assay and at 100 µg/mL in the DPPH• assay, and these two values were thereby used as the test concentrations during the radical scavenging assay.

**Table 2:** ABTS^•+^ and DPPH^•^ scavenging activity test results.

Samples	ABTS^• +^ Scavenging Activity (% Inhibition of 70 µg/mL± standard deviation)	DPPH^•^ Scavenging Activity (% Inhibition of 100 µg/mL ± standard deviation)
ShEO	83.973 ± 0.017 ^ab^	0.646 ± 0.197 ^e^
ShHME	37.732 ± 0.219 ^c^	19.748 ± 0.021 ^cd^
ShHWE	5.195 ± 0.224 ^g^	20.590 ± 0.002 ^cd^
ShRME	12.841 ± 0.018 ^f^	14.430 ± 0.023 ^d^
ShRWE	2.505 ± 0.007 ^g^	nd
SmEO	3.003 ± 0.004 ^g^	nd
SmHME	26.885 ± 0.028 ^d^	24.370 ± 0.027 ^c^
SmHWE	39.763 ± 0.015 ^c^	36.289 ± 0.027 ^b^
*α*- Tocopherol	19.569 ± 0.016 ^e^	90.225 ± 0.004 ^a^
Trolox	99.527 ± 0.004 ^a^	93.008 ± 0.001 ^a^

ShEO: *S. hortensis* essential oil*,* ShHME: *S. hortensis* herb methanol extract*,* ShHWE: *S. hortensis* herb water extract*,* ShRME *S. hortensis* root methanol extract*,* ShRWE: *S. hortensis* root water extract*,* SmEO: *S. montana* essential oil*,* SmHME *S. montana* methanol extract, SmHWE: *S. montana* water extract, nd: not detected. Samples sharing the same letter within a column are not significantly different from each other, whereas those with different letters show a significant statistical difference (p < 0.001); values labeled as “nd” (not determined) were excluded from the statistical analysis.

Among the tested plant materials, *Satureja hortensis* essential oil (ShEO) and *S. montana* herb water extract (SmHWE) emerged as the most active samples in the ABTS• ⁺ assay. ShEO demonstrated a remarkably high inhibition value of 83.973 ± 0.017%, surpassing even the reference antioxidant α-tocopherol, while Trolox exhibited near-complete inhibition (99.527 ± 0.004%). SmHWE followed with 39.763 ± 0.015% inhibition, indicating substantial radical scavenging capacity. Moderate ABTS• ⁺ activity was also observed in *S. hortensis* herb methanol extract (ShHME; 37.732 ± 0.219%) and *S. montana* herb methanol extract (SmHME; 26.885 ± 0.028%). In contrast, root-derived extracts (ShRME and ShRWE) and *S. montana* essential oil (SmEO) displayed significantly lower inhibition values than the reference standards.

A different distribution pattern was observed in the DPPH• assay. While Trolox (93.008 ± 0.001%) and α-tocopherol (90.225 ± 0.004%) showed the highest scavenging activities, SmHWE demonstrated the strongest inhibition (36.289 ± 0.027%) among the plant samples, followed by SmHME (24.370 ± 0.027%), ShHWE (20.590 ± 0.002%), and ShHME (19.748 ± 0.021%). In stark contrast to its ABTS• ⁺ performance, ShEO exhibited minimal DPPH• inhibition (0.646 ± 0.197%), and no activity was detected for SmEO and ShRWE.

Generally, methanolic and water extracts outperformed essential oils in both assays, with ShEO being the notable exception in the ABTS• ⁺ test. The root extracts of both species exhibited moderate activity, suggesting that both plant part and solvent polarity are influential factors in determining antioxidant efficacy. These results collectively indicate that herb-derived and water-extracted samples, particularly from *S. montana*, are the most promising sources of natural antioxidants among the tested materials.

### Enzyme inhibition assays

**Fig 2:**
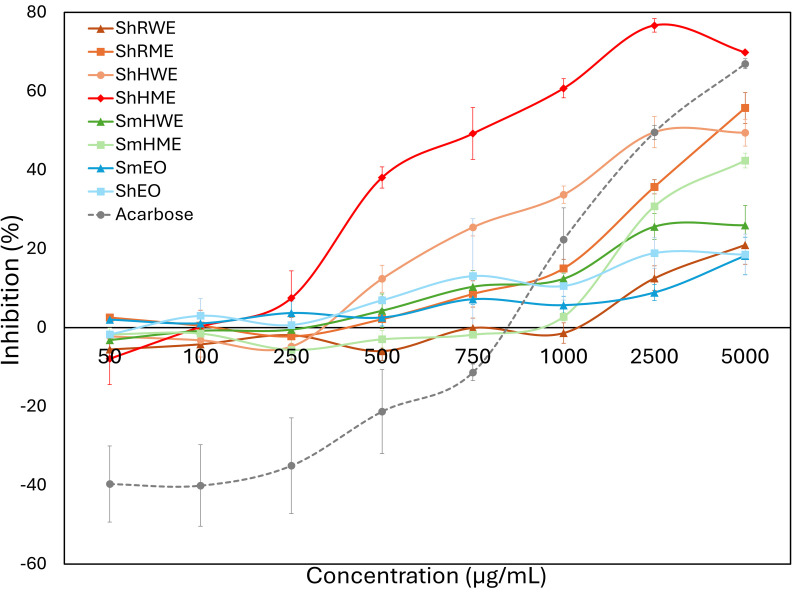
Concentration-Dependent *α*-glucosidase Inhibition by *S. hortensis* and *S. montana* Extracts and Essential Oils Compared to Acarbose.

**Fig 3:**
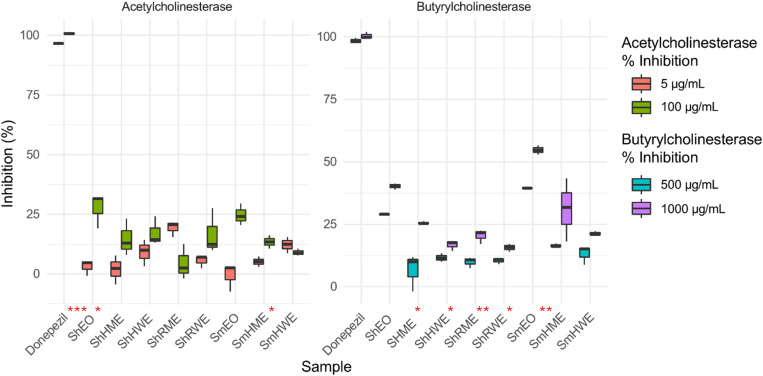
Inhibitory Effects of *S. hortensis* and *S. montana* Extracts on Acetylcholinesterase and Butyrylcholinesterase at Varying Concentrations (Asterisks on the plot indicate statistically significant differences between concentrations within each sample: * p < 0.05; ** p < 0.01; *** p < 0.001).

The enzyme inhibition results depicted in Fig 2 and 3 align well with the data presented in Tables 3 and 4, collectively demonstrating the distinct inhibitory potentials of *Satureja hortensis* and *Satureja montana* samples. Among the tested extracts, *S. hortensis* herb methanol extract (ShHME) exhibited the most prominent *α*-glucosidase inhibition (69.84%), closely approaching the inhibitory effect of the standard compound acarbose (66.95%) at 5000 µg/mL. Similarly, the root methanol extract (ShRME) and herb water extract (ShHWE) demonstrated notable inhibitory effects (55.73% and 49.45%, respectively).

**Table 3:** Antidiabetic activities results.

Antidiabetic Activities				
Samples	*α*-Glucosidase Inhibition (%) (5000 µg/mL) (mean ± std)	*α*-Glucosidase Inhibition IC_50_ (µg/mL)	*α*-Amylase Inhibition (%) (5000 µg/mL) (mean ± std)	*α*-Amylase Inhibition IC_50_ (µg/mL)
ShEO	18.53 ± 5.18 ^e^	–	ND	–
ShHME	69.84 ± 0.52 ^a^	785	ND	–
ShHWE	49.45 ± 3.42 ^c^	–	ND	–
ShRME	55.73 ± 3.90 ^b^	4237	ND	–
ShRWE	20.98 ± 4.91 ^e^	–	ND	–
SmEO	18.20 ± 4.74 ^e^	–	ND	–
SmHME	42.39 ± 1.85 ^cd^	–	ND	–
SmHWE	25.99 ± 4.98 ^de^	–	ND	–
Acarbose	66.95** **± 1.28 ^ab^	2313	59.65 ± 4.00	306

ShEO: *S. hortensis* essential oil*,* ShHME: *S. hortensis* herb methanol extract*,* ShHWE: *S. hortensis* herb water extract*,* ShRME *S. hortensis* root methanol extract*,* ShRWE: *S. hortensis* root water extract*,* SmEO: *S. montana* essential oil*,* SmHME *S. montana* methanol extract, SmHWE: *S. montana* water extract, nd: not detected. Samples sharing the same letter within a column are not significantly different from each other, whereas those with different letters show a significant statistical difference (p < 0.001); values labeled as “nd” (not determined) were excluded from the statistical analysis. † IC_50_ values are reported only for extracts or oils exhibiting >50% inhibition at the tested concentration range. Samples below this threshold were not analyzed for IC_50_.

**Table 4:** Anticholinesterase activities results.

			Anticholinesterase Activities		
Samples	Acetylcholinesterase Inhibition (%) (100 µg/mL) (mean ± std)	AChE IC_50_ (µg/mL)		Butyrylcholinesterase Inhibition (%) (1000 µg/mL) (mean ± std)	BchE IC_50_ (µg/mL)
ShEO	31.85 ± 0.39 ^b^	–		40.20 ± 1.24 ^c^	–
ShHME	10.46 ± 3.56 ^d^	–		25.58 ± 0.64 ^d^	–
ShHWE	13.78 ± 0.87 ^cd^	–		16.73 ± 2.06 ^e^	–
ShRME	19.28 ± 3.24 ^c^	–		20.43 ± 2.83 ^de^	–
ShRWE	11.21 ± 1.76 ^d^	–		15.71 ± 1.59 ^e^	–
SmEO	24.74 ± 4.63 ^bc^	–		54.71 ± 1.82 ^b^	76.39
SmHME	13.45 ± 2.70 ^cd^	–		37.58 ± 8.27 ^c^	–
SmHWE	9.26 ± 2.02 ^d^	–		21.35 ± 0.85 ^de^	–
Donepezil	100 ± 0.55 ^a^	0.29		100 ± 1.38 ^a^	15.39

ShEO: *S. hortensis* essential oil*,* ShHME: *S. hortensis* herb methanol extract*,* ShHWE: *S. hortensis* herb water extract*,* ShRME *S. hortensis* root methanol extract*,* ShRWE: *S. hortensis* root water extract*,* SmEO: *S. montana* essential oil*,* SmHME *S. montana* methanol extract, SmHWE: *S. montana* water extract, nd: not detected. Samples sharing the same letter within a column are not significantly different from each other, whereas those with different letters show a significant statistical difference (p < 0.001); values labeled as “nd” (not determined) were excluded from the statistical analysis. † IC_50_ values are reported only for extracts or oils exhibiting >50% inhibition at the tested concentration range. Samples below this threshold were not analyzed for IC_50_.

Although *α*-amylase inhibition was evaluated across a wide concentration range (1–5000 µg/mL), none of the tested samples exhibited measurable activity, and inhibition values remained below the detection limit. This lack of effect may be related to the low content or absence of α-amylase-interacting phytochemicals in the extracts.

With the increasing concentration, most extracts showed an increasing trend of inhibition on *α*-glucosidase activity. Notably, the inhibition from ShHME started to increase at a concentration of 250 µg/mL and maintained at a significantly higher level than the reference acarbose throughout the entire concentration gradient (Fig 2). The extract ShHWE also stood out with significantly higher inhibition than acarbose at a concentration range of 500–1000 µg/mL (Fig 2). Conversely, none of the tested samples showed detectable α-amylase inhibition (Table 3). Regarding cholinesterase inhibition (Fig 3), the essential oil of *S. hortensis* (ShEO) showed the highest activity against acetylcholinesterase (31.85 ± 0.39%), while *S. montana* essential oil (SmEO) was the most effective inhibitor of butyrylcholinesterase (54.71 ± 1.82%) at 1000 µg/mL. Notably, SmHME and ShEO also demonstrated substantial BChE inhibition (37.58% and 40.20%, respectively), suggesting that volatile constituents may play a significant role in cholinesterase inhibition. The high *α*-glucosidase inhibitory potential of *S. hortensis* methanol extracts and the strong BChE inhibition by essential oils particularly from *S. Montana* observed here implies their potential application in the management of type 2 diabetes and neurodegenerative disorders.

### Correlation and multivariate analysis of enzyme inhibition and antioxidant activities in *Satureja* extracts and essential oils

**Fig 4:**
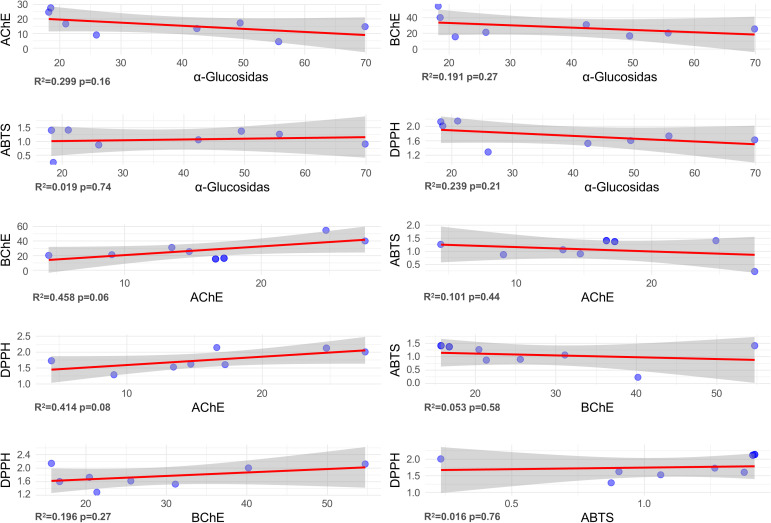
Correlation analysis between enzyme inhibition activities and antioxidant assays.

Pairwise correlations among the biological activities of various *Satureja* extracts and essential oils including enzyme inhibitory activities (α-glucosidase, acetylcholinesterase (AChE), and butyrylcholinesterase (BChE)) and antioxidant capacities (ABTS•⁺ and DPPH• radical scavenging) are presented in Fig 4. Although several correlations were statistically weak or non-significant, the emerging trends offer biologically meaningful insights into the phytochemical landscape and possible therapeutic implications of these plant-based extracts.

A weak to moderate inverse relationship was observed between α-glucosidase inhibition and cholinesterase (AChE and BChE) inhibitory activities (R² = 0.299 and 0.191, respectively). This trend suggests that extracts exhibiting stronger antidiabetic potential via α-glucosidase inhibition tend to exhibit lower neuroprotective potential via cholinesterase inhibition, and vice versa. While these associations were not statistically robust, the consistent negative trends may reflect differences in the phytochemical profiles driving these activities potentially indicating that distinct classes of bioactive compounds are responsible for targeting each enzyme system.

In contrast, the correlations between α-glucosidase inhibition and antioxidant assays (ABTS•⁺ and DPPH•) were minimal or inconsistent, with low R² values (0.019 and 0.239, respectively), further reinforcing the idea that glucosidase inhibition is largely independent of free radical scavenging activity. This divergence suggests that the compounds responsible for α-glucosidase inhibition possibly non-phenolic or enzyme-specific small molecules may not necessarily possess antioxidant properties, and may act through different mechanisms.

A moderate positive correlation was identified between AChE and BChE inhibition (R² = 0.458), implying a degree of functional overlap in extracts capable of dual cholinesterase inhibition. Such co-inhibition may arise from shared neuroactive constituents such as phenolic diterpenes or flavonoids. However, the potency and ratio of inhibition for each enzyme still varied across samples, indicating differential affinities and selectivities among the constituent compounds.

Interestingly, the relationship between cholinesterase inhibition and antioxidant capacity was notably weak across all extract types. This lack of correlation suggests that neuroprotective and antioxidant activities are mediated by chemically and functionally distinct sets of phytochemicals. Such functional decoupling has been reported previously in polyphenol-rich plants, where structural differences (e.g., hydroxylation pattern, glycosylation) can lead to divergent bioactivity profiles.

As expected, ABTS•⁺ and DPPH• assays, both of which assess radical scavenging potential, demonstrated a weak positive correlation (R² = 0.196), indicating partial overlap in the antioxidant compounds detectable by each assay. The relatively low correlation could be attributed to the differences in reaction mechanisms ABTS being more sensitive to hydrophilic antioxidants and DPPH more responsive to lipophilic species as well as variations in extraction solvent polarity, which influence the phytochemical composition of the extracts.

**Fig 5:**
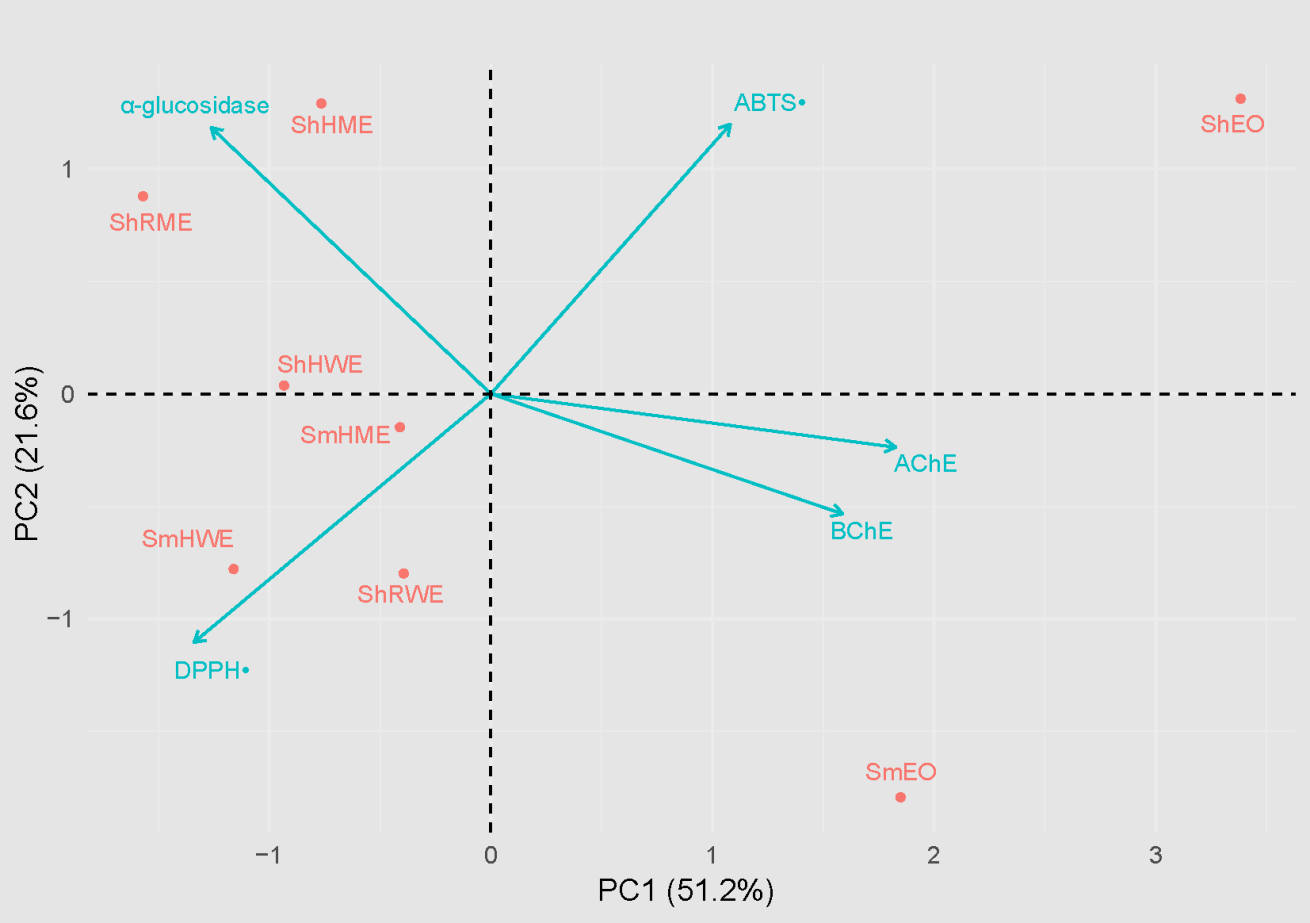
Principal Component Analysis (PCA) biplot of bioactivities of *Satureja* extracts and essential oils.

To better understand the multivariate relationships among the extracts and their bioactivities, a Principal Component Analysis (PCA) was performed (Fig 5). The first two principal components (PC1 and PC2) accounted for 51.2% and 21.6% of the total variance, respectively, effectively capturing the majority of biological variability across the samples. The PCA biplot revealed distinct clustering patterns among the *Satureja* samples, reflecting clear differences in their pharmacological profiles.

For instance, ShHME and ShRME were positioned along the direction of the α-glucosidase vector, suggesting that these extracts are enriched in compounds with strong antidiabetic potential. In contrast, SmEO and ShEO, both essential oil samples, were located along the positive end of PC1 and showed strong alignment with the AChE and BChE vectors. This indicates that volatile oil constituents such as monoterpenes and sesquiterpenes might be responsible for the observed cholinesterase inhibition. These findings support the hypothesis that essential oils, due to their lipophilic nature and unique chemical composition, may be more potent against cholinesterase targets compared to polar extracts.

Moreover, ShHWE, SmHME, and SmHWE were associated with the DPPH• vector, suggesting elevated antioxidant potential in these water-based and hydroethanolic extracts, likely due to a higher content of hydrophilic polyphenols. The orientation and opposition of vectors in the biplot further revealed functional trade-offs. Notably, the ABTS•⁺ and DPPH• vectors pointed in opposing directions, implying a negative correlation between the two antioxidant assays within this dataset. This result is consistent with the differential sensitivity of the assays to distinct antioxidant types and underscores the complexity of antioxidant profiling in multi-component plant extracts. Similarly, the inverse orientation of α-glucosidase and cholinesterase vectors reflects the negative correlation previously observed in the pairwise plots, suggesting that phytochemical constituents may have selective inhibitory effects that favor one pathway over another.

Taken together, these multivariate analyses highlight the functional differentiation among *Satureja* extracts based on their extraction method and phytochemical composition. The results suggest that specific extracts may be tailored for targeted therapeutic purposes such as antidiabetic or neuroprotective applications depending on their bioactivity profile. This functional specificity underscores the importance of choosing appropriate extraction techniques and underscores the value of integrative statistical tools like PCA in unraveling complex biological data.

## Discussion

Despite the fact that the phytochemical profiles of *Satureja hortensis* and *Satureja montana* have been extensively investigated, the concentrations can be affected by various factors, e.g., plant growth condition, oil extraction protocol, genotypic variation and chemical determination method. In *S. hortensis*, carvacrol (67%), γ-terpinene (15.3%), and p-cymene (6.73%) have been identified as predominant constituents [31], while another study reported carvacrol (33.7%) and γ-terpinene (31.8%) as the major compounds in oils obtained from air-dried plant material [32]. More than 40 volatile components have been identified in the essential oil, among which carvacrol, 1,8-cineole, eugenol, β-humulene, linalool, β-pinene, α-terpineol, and thymol have demonstrated significant antibacterial activity against several pathogenic bacterial genera [33]. Thus, to select *Satureja* varieties with a maximum medicinal value, this study thoroughly investigated the phytochemical profiles of two types of *Satureja*, particularly in relation to the composition and bioactivity of their essential oils. Our results in *S. hortensis*, e.g., carvacrol (49.0%), γ-terpinene (34.3%), and p-cymene (3.1%) align well with previous reports. The drying and distillation methods used prior to oil extraction significantly influence its chemical composition. Hydro-distillation of aerial parts yielded the highest oil content (0.94%) and carvacrol levels (46.0–48.1%), whereas steam-distillation of shade-dried samples led to a notable decrease in carvacrol (12.3%) and increase in γ-terpinene (70.4%) [11].

Similarly, *S. montana* essential oil has been shown to contain a complex mixture of monoterpenoids and phenolic compounds. GC-MS analysis identified 20 constituents accounting for 97% of the total oil, with carvacrol (45.7%), p-cymene, γ-terpinene, carvacrol methyl ether, and thymol as major components [34]. Essential oils from dried stems were composed predominantly of monoterpenoids (84.4–97.6%), with carvacrol ranging between 82.5 and 95.0% [35]. Plants cultivated in northeastern Romania produced oils rich in carvacrol (63.40%), p-cymene (10.97%), and γ-terpinene (3.70%) [36]. These reported concentrations are in line with our present observations, e.g., carvacrol (54.4%), p-cymene (21.9%) and γ-terpinene (4.4%). The consistency of these findings across different geographical origins and processing methods supports the chemotaxonomic reliability of both species.

Building upon the established chemical profiles of *Satureja hortensis* and *Satureja montana*, their antioxidant capacities were further evaluated using two widely adopted spectrophotometric methods: 2,2-azino-bis-3-ethylbenzthiazoline-6-sulfonic acid (ABTS•⁺) and 1,1-diphenyl-2-picrylhydrazyl (DPPH•). These electron-transfer-based assays offer key advantages, including simplicity, rapid response, and suitability across both hydrophilic and lipophilic environments. Nevertheless, the use of non-physiological radicals in both methods may present limitations, particularly in food system evaluations. The ABTS assay, which generates the blue-green ABTS⁺ radical cation, is applicable in both polar and nonpolar systems, while the DPPH method is more appropriate for lipophilic matrices due to its use of a radical dissolved in organic media [37–39].

To assess the influence of extraction solvents on antioxidant efficacy, both ABTS•⁺ and DPPH• assays were applied to various extracts and essential oils. The results from both methods were generally in agreement; however, the essential oil of *S. hortensis* (ShEO) demonstrated notably stronger activity in the ABTS• ⁺ assay compared to the reference standard α-tocopherol. This superior performance may result from the enhanced interaction of ShEO’s terpenoid constituents with the ABTS• ⁺ radical, enabling more efficient radical quenching. Supporting evidence was provided by Alan et al. [40], who reported 87.2% ABTS• ⁺ radical scavenging activity at 100 µg/mL for *S. hortensis* essential oil, which exceeded the effect of α-tocopherol. This activity has been linked to the presence of bioactive terpenes, particularly monoterpenes like carvacrol and thymol [41].

Comparative assessments in *S. montana* have yielded similar insights. Serrano et al. [14] reported DPPH• radical scavenging activity for cold water, hot water, ethanolic extracts, and essential oils, with IC₅₀ values ranging from 9.8 to 508.5 µg/mL. The essential oil displayed the lowest antioxidant efficacy among the tested forms. In contrast, Ćavar et al. [42] measured the DPPH• IC₅₀ value of *S. montana* essential oil as 5.49 mg/mL and 18.9 mg/mL for two different batches, while thymol showed a reference value of 13.3 mg/mL. For *S. subspicata*, the values were even higher, at 25.6 mg/mL and 21.0 mg/mL, respectively.

Overall, the compiled data confirm that both *S. hortensis* and *S. montana* exhibit considerable antioxidant potential, which is closely associated with their terpenoid and phenolic content. The high activity observed in selected extracts and essential oils underscores the importance of extraction technique and phytochemical profile in determining antioxidant efficacy, and further supports their potential as natural sources of functional antioxidant agents.

A study evaluated the *α*-amylase inhibitory activity of *S. hortensis* extracts obtained using different extraction techniques (traditional, ultrasound-assisted, and microwave-assisted) and ethanol concentrations (40%, 50%, 60%). All extracts demonstrated inhibitory effects against *α*-amylase at a concentration of 400 µg/mL, with inhibition values ranging from 8.35% to 50.77%, depending on the method and solvent used. The highest *α*-amylase inhibition (49.51%) was observed with ultrasound-assisted extraction for 30 minutes using 60% ethanol [42]. In another study, the essential oil of *S. hortensis* (SEO) was evaluated and exhibited potent *α*-amylase inhibitory activity with an IC₅₀ of 230 µg/mL. The anti-hyperglycemic potential of the essential oil was also supported by molecular evidence SEO reduced NOX and NF-κB expression while enhancing NRF2 expression in hyperglycemia-induced macrophages. The activity was attributed to its high content of carvacrol, thymol, and γ-terpinene, suggesting a synergistic inhibitory mechanism involving phenolic monoterpenes [43]. Another study evaluated the anti-amylase effect of fatty acids extracted from *S. hortensis*. The plant’s fatty acids demonstrated significant *α*-amylase inhibition, with an IC₅₀ range of 370–390 µg/mL, and an inhibitory capacity equivalent to 752–790 mg acarbose/g fatty acid. These values correspond to 68.36–71.80 mg acarbose equivalent per gram of *Satureja* dried powder. Kinetic analyses showed that *S. hortensis* fatty acids decreased the Vmax of amylase without significantly affecting the Km, indicating an uncompetitive inhibition mechanism. The activity is attributed to its rich composition of polyunsaturated fatty acids (especially omega-3 and omega-6), monoterpenes (thymol, carvacrol), and other lipophilic bioactives. Spectroscopic studies also confirmed the binding of fatty acids to the enzyme, causing conformational changes and enzyme inhibition [44]. In a study, the ethanolic extract of *S. hortensis* exhibited strong *α*-glucosidase inhibition, with an inhibition rate of 96.12% at 1000 ppm and an IC₅₀ value of 29 ppm, indicating potent antidiabetic potential. However, its *α*-amylase inhibition was low (<50%), and thus its IC₅₀ for this enzyme was not determined in detail [45].

In a study, among 18 Lamiaceae species tested, the aqueous extract of *S. montana* showed strong *α*-glucosidase inhibitory activity, achieving 89.38% inhibition at 0.5 mg/mL, which was notably higher than that of the reference standard acarbose (71.35%). Even at lower concentrations (0.25 mg/mL and 0.1 mg/mL), it demonstrated substantial inhibition (72.11% and 38.41%, respectively). This extract outperformed both methanolic and ethanolic counterparts in activity. The LC-MS profiling revealed salvianolic acid A as the dominant phenolic acid (21.483 mg/L) in the methanolic extract of *S. montana*, and molecular docking simulations confirmed high binding affinities of its constituents (e.g., flavonoids and phenolic acids) to both yeast and human *α*-glucosidase. These compounds interact with both active and allosteric sites, contributing to the plant’s potent and multi-site enzyme inhibition [45].

In the present study, *S. hortensis* herb methanol extract (ShHME) demonstrated the most prominent *α*-glucosidase inhibitory activity (69.84% at 5000 µg/mL), comparable to the standard acarbose (66.95%). Similarly, root methanol (ShRME, 55.73%) and herb water (ShHWE, 49.45%) extracts also showed strong inhibitory effects. These findings align partially with the study by Zarei et al. [45], which reported even higher inhibition (96.12% at 1000 ppm) for ethanolic *S. hortensis* extract. However, in contrast to previous reports where *S. hortensis* also exhibited moderate to strong *α*-amylase inhibition (e.g., 49.51% using ultrasound-assisted extraction with 60% ethanol; [46]), none of our samples showed detectable *α*-amylase inhibitory activity. This discrepancy may be due to differences in extraction methods, solvent polarity, or sample composition. Regarding *S. montana*, although earlier research by Pavlović et al. [47] reported stronger *α*-glucosidase inhibition (up to 89.38% at 0.5 mg/mL) by the aqueous extract, our results revealed moderate activity from the methanol and water extracts, suggesting that solvent type and phytochemical composition critically influence bioactivity. Collectively, our findings highlight the remarkable *α*-glucosidase inhibitory potential of *S. hortensis*, particularly in polar extracts, and support its utility in managing postprandial hyperglycemia.

In a study, the anticholinesterase activity of *S. montana* was evaluated using extracts obtained via conventional methods (hydrodistillation and soxhlet extraction) and supercritical fluid extraction. The volatile fractions, particularly the essential oil obtained by hydrodistillation, exhibited strong dual inhibition against acetylcholinesterase (AChE) and butyrylcholinesterase (BChE), with IC₅₀ values of 45 µg/mL and 52 µg/mL, respectively. This effect was primarily attributed to the high levels of carvacrol and thymol present in the oil. The SFE volatile extract also inhibited both enzymes but to a slightly lesser extent. Interestingly, the nonvolatile fractions obtained via SFE (especially extract B2) selectively inhibited BChE (IC₅₀: 34 µg/mL), whereas the Soxhlet acetone extract showed no significant activity on either enzyme. The selective BChE inhibition in the SFE extracts was linked to the presence of phenolic compounds such as catechin, vanillic acid, chlorogenic acid, and protocatechuic acid [23]. In a comparative study evaluating the acetylcholinesterase (AChE) inhibitory activity of 26 medicinal plants from the Lamiaceae family, *S. montana* exhibited strong, dose-dependent inhibition. At a concentration of 1 mg/mL, the ethanolic extract of *S. montana* achieved AChE inhibition above 75%, placing it among the most potent species tested, alongside *Mentha x piperita*, *Salvia officinalis*, and *Thymus vulgaris*. The extract also showed considerable antioxidant activity (IC₅₀ = 9.95 µg/mL; total antioxidant capacity: 717.41 mg TE/g). High levels of rosmarinic acid (31.11 mg/g extract) and total hydroxycinnamic acid derivatives (95.29 mg/g) were identified, which are thought to contribute significantly to both AChE inhibition and antioxidant effects [48]. In another study, the essential oils of *S. montana* ssp. *montana*, collected from three different altitudes (100 m, 500 m, and 800 m), were evaluated for their anticholinesterase activity alongside antioxidant and antimicrobial effects. All three oils significantly inhibited human serum cholinesterase activity, with the strongest effect observed in the oil from the lowest altitude (S1), which exhibited 85.71% inhibition, followed by S2 (51.16%) and S3 (49.17%). The high activity of the S1 oil was attributed to its rich content of phenolic monoterpenes, particularly thymol (24.69%) and carvacrol (15.19%), whereas oils from higher altitudes contained lower amounts of these compounds and higher concentrations of alcohols such as linalool and terpinen-4-ol [49].

This study provides the first comprehensive evaluation of the anticholinesterase activity of *S. hortensis*, revealing that its essential oil (ShEO) exhibited the strongest acetylcholinesterase inhibition (31.85%) and notable butyrylcholinesterase inhibition (40.20%) at the tested concentrations. While no prior study has specifically assessed the cholinesterase inhibitory effects of *S. hortensis*, our results suggest that phenolic monoterpenes such as carvacrol and *γ*-terpinene may play a key role. In comparison, *S. montana* essential oil (SmEO) showed the highest BChE inhibition (54.71%), corroborating findings from Silva et al. [23], who reported dual AChE and BChE inhibition by hydrodistilled essential oil (IC₅₀: 45 µg/mL for AChE, 52 µg/mL for BChE), attributed to carvacrol and thymol content. Similarly, Mihajilov-Krstev et al. [49] found up to 85.71% cholinesterase inhibition in SmEO from low altitudes, again emphasizing the role of phenolic constituents. Moreover, the ethanolic extract of *S. montana* displayed strong, dose-dependent AChE inhibition (>75%) in another comparative study [23], linked to rosmarinic acid and hydroxycinnamic derivatives. Although our *S. montana* extracts showed moderate cholinesterase inhibition, the essential oil distinctly outperformed the extracts. Overall, the anticholinesterase findings reinforce the therapeutic potential of *Satureja* species, particularly *S. montana* oil as a potent BChE inhibitor and *S. hortensis* oil as a novel AChE-active agent.

## Conclusion

This study comprehensively evaluated the *in vitro* antidiabetic, antioxidant, and cholinesterase inhibitory properties of extracts and essential oils derived from *Satureja hortensis* and *Satureja montana*. The methanol extract of *S. hortensis* aerial parts (ShHME) exhibited the most prominent *α*-glucosidase inhibitory activity, comparable to the reference compound acarbose. Water and root extracts also demonstrated considerable enzyme inhibition, highlighting the significance of both plant part and solvent polarity in determining bioactivity. Although no *α*-amylase inhibition was detected, the consistent *α*-glucosidase inhibitory results, particularly from polar extracts, indicate promising potential for further investigation.

In terms of antioxidant capacity, *S. hortensis* essential oil (ShEO) showed remarkable ABTS• ⁺ radical scavenging activity, even surpassing *α*-tocopherol, while the water extract of *S. montana* (SmHWE) displayed the highest activity in the DPPH• assay. These findings suggest that both volatile and polar constituents contribute to antioxidant performance, with monoterpenes such as carvacrol and thymol likely playing a key role.

With regard to cholinesterase inhibition, *S. montana* essential oil (SmEO) showed the strongest BChE inhibitory effect, while *S. hortensis* oil (ShEO) demonstrated the highest AChE inhibition. GC-MS analysis confirmed the presence of phenolic monoterpenes, which may account for these neuroprotective effects. Multivariate statistical analysis (PCA) revealed a clear functional distinction between the extract and essential oil groups: extracts were more closely associated with *α*-glucosidase inhibition and antioxidant activity, while essential oils clustered with cholinesterase inhibitory effects.

Overall, the findings of this study provide evidence supporting the pharmacological potential of *Satureja hortensis* and *Satureja montana*, particularly as multifunctional botanical candidates with antioxidant, antidiabetic, and neuroprotective properties. While the results offer valuable insight into the biological profiles of these culinary herbs, additional in-depth studies, especially in vivo and mechanistic investigations are needed to better understand their modes of action and to validate their relevance for therapeutic development.

## Supporting information

S1 Table Data for DPPH radical scavenging activity. (XLSX)

S2 Table Data for ABTS radical scavenging activity. (XLSX)

S3 Table Data for α-Glucosidase inhibitory activity. (XLSX)

S4 Table Data for α-Amylase inhibitory activity assay. (XLSX)

S5 Table Data for Acetylcholinesterase (AChE) inhibitory activities. (XLSX)

S6 Table Data for Butyrylcholinesterase (BChE) inhibitory activities. (XLSX)

## References

[1] Alam S, Hasan MdK, Neaz S, Hussain N, Hossain MdF, Rahman T. Diabetes Mellitus: Insights from Epidemiology, Biochemistry, Risk Factors, Diagnosis, Complications and Comprehensive Management. Diabetology. 2021;2(2):36–50. doi: 10.3390/diabetology2020004

[2] Sims-Robinson C, Kim B, Rosko A, Feldman EL. How does diabetes accelerate Alzheimer disease pathology?. Nat Rev Neurol. 2010;6(10):551–9. doi: 10.1038/nrneurol.2010.130 20842183 PMC3199576

[3] Korczyn AD, Grinberg LT. Is Alzheimer disease a disease? Nature Reviews Neurology. 2024;20(4):245–51. 38424454 10.1038/s41582-024-00940-4 PMC12077607

[4] Patil RS, Tupe RS. Communal interaction of glycation and gut microbes in diabetes mellitus, Alzheimer’s disease, and Parkinson’s disease pathogenesis. Med Res Rev. 2024;44(1):365–405. doi: 10.1002/med.21987 37589449

[5] Ahtiluoto S, Polvikoski T, Peltonen M, Solomon A, Tuomilehto J, Winblad B, et al. Diabetes, Alzheimer disease, and vascular dementia: a population-based neuropathologic study. Neurology. 2010;75(13):1195–202. doi: 10.1212/WNL.0b013e3181f4d7f8 20739645

[6] Huang J, Huang N, Mao Q, Shi J, Qiu Y. Natural bioactive compounds in Alzheimer’s disease: From the perspective of type 3 diabetes mellitus. Front Aging Neurosci. 2023;15:1130253. doi: 10.3389/fnagi.2023.1130253 37009462 PMC10062602

[7] Shrivastav D, Dabla PK, Sharma J, Viswas A, Mir R. Insights on antioxidant therapeutic strategies in type 2 diabetes mellitus: A narrative review of randomized control trials. World J Diabetes. 2023;14(6):919–29. doi: 10.4239/wjd.v14.i6.919 37383600 PMC10294058

[8] Park JS, Rehman IU, Choe K, Ahmad R, Lee HJ, Kim MO. A Triterpenoid Lupeol as an Antioxidant and Anti-Neuroinflammatory Agent: Impacts on Oxidative Stress in Alzheimer’s Disease. Nutrients. 2023;15(13):3059. doi: 10.3390/nu15133059 37447385 PMC10347110

[9] Infante-Garcia C, Garcia-Alloza M. Review of the Effect of Natural Compounds and Extracts on Neurodegeneration in Animal Models of Diabetes Mellitus. Int J Mol Sci. 2019;20(10):2533. doi: 10.3390/ijms20102533 31126031 PMC6566911

[10] Sahin F, Karaman I, Güllüce M, Oğütçü H, Sengül M, Adigüzel A, et al. Evaluation of antimicrobial activities of Satureja hortensis L. J Ethnopharmacol. 2003;87(1):61–5. doi: 10.1016/s0378-8741(03)00110-7 12787955

[11] Sefidkon F, Abbasi K, Khaniki GB. Influence of drying and extraction methods on yield and chemical composition of the essential oil of Satureja hortensis. Food Chemistry. 2006;99(1):19–23. doi: 10.1016/j.foodchem.2005.07.026

[12] Fierascu I, Dinu-Pirvu CE, Fierascu RC, Velescu BS, Anuta V, Ortan A, et al. Phytochemical Profile and Biological Activities of Satureja hortensis L.: A Review of the Last Decade. Molecules. 2018;23(10):2458. doi: 10.3390/molecules23102458 30257512 PMC6222901

[13] Slavkovska V, Jancic R, Bojovic S, Milosavljevic S, Djokovic D. Variability of essential oils of Satureja montana L. and Satureja kitaibelii wierzb. ex Heuff. from the central part of the balkan peninsula. Phytochemistry. 2001;57(1):71–6. doi: 10.1016/s0031-9422(00)00458-1 11336264

[14] Serrano C, Matos O, Teixeira B, Ramos C, Neng N, Nogueira J, et al. Antioxidant and antimicrobial activity of Satureja montana L. extracts. J Sci Food Agric. 2011;91(9):1554–60. doi: 10.1002/jsfa.4347 21445865

[15] Zavatti M, Zanoli P, Benelli A, Rivasi M, Baraldi C, Baraldi M. Experimental study on Satureja montana as a treatment for premature ejaculation. J Ethnopharmacol. 2011;133(2):629–33. doi: 10.1016/j.jep.2010.10.058 21040774

[16] Kumburovic I, Selakovic D, Juric T, Jovicic N, Mihailovic V, Stankovic JK, et al. Antioxidant Effects of Satureja hortensis L. Attenuate the Anxiogenic Effect of Cisplatin in Rats. Oxid Med Cell Longev. 2019;2019:8307196. doi: 10.1155/2019/8307196 31467638 PMC6701305

[17] Bezić N, Samanić I, Dunkić V, Besendorfer V, Puizina J. Essential oil composition and internal transcribed spacer (ITS) sequence variability of four South-Croatian Satureja species (Lamiaceae). Molecules. 2009;14(3):925–38. doi: 10.3390/molecules14030925 19255551 PMC6253779

[18] Hamidpour R, Hamidpour S, Hamidpour M, Shahlari M, Sohraby M. Summer Savory: From the Selection of Traditional Applications to the Novel Effect in Relief, Prevention, and Treatment of a Number of Serious Illnesses such as Diabetes, Cardiovascular Disease, Alzheimer’s Disease, and Cancer. J Tradit Complement Med. 2014;4(3):140–4. doi: 10.4103/2225-4110.136540 25161917 PMC4142450

[19] Hajhashemi V, Ghannadi A, Pezeshkian SK. Antinociceptive and anti-inflammatory effects of Satureja hortensis L. extracts and essential oil. J Ethnopharmacol. 2002;82(2–3):83–7. doi: 10.1016/s0378-8741(02)00137-x 12241981

[20] Hudz N, Makowicz E, Shanaida M, Białoń M, Jasicka-Misiak I, Yezerska O, et al. Phytochemical Evaluation of Tinctures and Essential Oil Obtained from Satureja montana Herb. Molecules. 2020;25(20):4763. doi: 10.3390/molecules25204763 33081385 PMC7587570

[21] Les F, Galiffa V, Cásedas G, Moliner C, Maggi F, López V, et al. Essential Oils of Two Subspecies of Satureja montana L. against Gastrointestinal Parasite Anisakis simplex and Acetylcholinesterase Inhibition. Molecules. 2024;29(19):4640. doi: 10.3390/molecules29194640 39407570 PMC11477606

[22] Grosso C, Figueiredo AC, Burillo J, Mainar AM, Urieta JS, Barroso JG, et al. Enrichment of the thymoquinone content in volatile oil from Satureja montana using supercritical fluid extraction. J Sep Sci. 2009;32(2):328–34. doi: 10.1002/jssc.200800490 19156634

[23] Silva FVM, Martins A, Salta J, Neng NR, Nogueira JMF, Mira D, et al. Phytochemical profile and anticholinesterase and antimicrobial activities of supercritical versus conventional extracts of Satureja montana. J Agric Food Chem. 2009;57(24):11557–63. doi: 10.1021/jf901786p 19928761

[24] Karakaya S, Yuca H, Yılmaz G, Aydın B, Tekman E, Ekşi G, et al. Phytochemical screening, biological evaluation, anatomical, and morphological investigation of Ferula tingitana L. (Apiaceae). Protoplasma. 2023;260(6):1581–601. doi: 10.1007/s00709-023-01874-2 37338647

[25] Blois MS. Antioxidant Determinations by the Use of a Stable Free Radical. Nature. 1958;181(4617):1199–200. doi: 10.1038/1811199a0

[26] Aydın B, Yuca H, Karakaya S, Bona GE, Göger G, Tekman E, et al. The anatomical, morphological features, and biological activity of Scilla siberica subsp. armena (Grossh.) Mordak (Asparagaceae). Protoplasma. 2023;260(2):371–89. doi: 10.1007/s00709-022-01784-9 35716225

[27] Re R, Pellegrini N, Proteggente A, Pannala A, Yang M, Rice-Evans C. Antioxidant activity applying an improved ABTS radical cation decolorization assay. Free Radic Biol Med. 1999;26(9–10):1231–7. doi: 10.1016/s0891-5849(98)00315-3 10381194

[28] Bachhawat JA, Shihabudeen MS, Thirumurugan K. Screening of fifteen Indian ayurvedic plants for alpha-glucosidase inhibitory activity and enzyme kinetics. International Journal of Pharmacy and Pharmaceutical Sciences. 2011;3:267–74.

[29] Nampoothiri SV, Prathapan A, Cherian OL, Raghu KG, Venugopalan VV, Sundaresan A. In vitro antioxidant and inhibitory potential of Terminalia bellerica and Emblica officinalis fruits against LDL oxidation and key enzymes linked to type 2 diabetes. Food Chem Toxicol. 2011;49(1):125–31. doi: 10.1016/j.fct.2010.10.006 20951180

[30] Ingkaninan K, de Best CM, van der Heijden R, Hofte AJ, Karabatak B, Irth H, et al. High-performance liquid chromatography with on-line coupled UV, mass spectrometric and biochemical detection for identification of acetylcholinesterase inhibitors from natural products. J Chromatogr A. 2000;872(1–2):61–73. doi: 10.1016/s0021-9673(99)01292-3 10749487

[31] Mihajilov-Krstev T, Radnović D, Kitić D, Zlatković B, Ristić M, Branković S. Chemical composition and antimicrobial activity of Satureja hortensis L. essential oil. Open Life Sciences. 2009;4(3):411–6. doi: 10.2478/s11535-009-0027-z 21922928

[32] Hajhashemi V, Sadraei H, Ghannadi AR, Mohseni M. Antispasmodic and anti-diarrhoeal effect of Satureja hortensis L. essential oil. J Ethnopharmacol. 2000;71(1–2):187–92. doi: 10.1016/s0378-8741(99)00209-3 10904162

[33] Deans SG, Svoboda KP. Antibacterial activity of summer savory(Satureja hortensis L)essential oil and its constituents. Journal of Horticultural Science. 1989;64(2):205–10. doi: 10.1080/14620316.1989.11515946

[34] Bezić N, Skočibušić M, Dunkić V. Phytochemical composition and antimicrobial activity of Satureja montana L. and Satureja cuneifolia Ten. essential oils. Acta Botanica Croatica. 2005;64(2):313–22.

[35] Santos JDC, Coelho E, Silva R, Passos CP, Teixeira P, Henriques I, et al. Chemical composition and antimicrobial activity of Satureja montana byproducts essential oils. Industrial Crops and Products. 2019;137:541–8. doi: 10.1016/j.indcrop.2019.05.058

[36] Trifan A, Aprotosoaie AC, Brebu M, Cioancă O, Gille E, Hăncianu M, et al. Chemical composition and antioxidant activity of essential oil from Romanian Satureja montana L. Farmacia. 2015;63(3):413–6.

[37] Floegel A, Kim D-O, Chung S-J, Koo SI, Chun OK. Comparison of ABTS/DPPH assays to measure antioxidant capacity in popular antioxidant-rich US foods. Journal of Food Composition and Analysis. 2011;24(7):1043–8. doi: 10.1016/j.jfca.2011.01.008

[38] Bibi Sadeer N, Montesano D, Albrizio S, Zengin G, Mahomoodally MF. The Versatility of Antioxidant Assays in Food Science and Safety-Chemistry, Applications, Strengths, and Limitations. Antioxidants (Basel). 2020;9(8):709. doi: 10.3390/antiox9080709 32764410 PMC7464350

[39] Schaich KM, Tian X, Xie J. Reprint of “Hurdles and pitfalls in measuring antioxidant efficacy: A critical evaluation of ABTS, DPPH, and ORAC assays”. Journal of Functional Foods. 2015;18:782–96. doi: 10.1016/j.jff.2015.05.024

[40] Alan Y, Savcı A, Çakmak B, Kurt H. Determination of the antimicrobial and antioxidant activities of Satureja hortensis ingredients. Yüzüncü Yıl Üniversitesi Fen Bilimleri Enstitüsü Dergisi. 2016;21(2):167–77.

[41] Torres-Martínez R, García-Rodríguez YM, Ríos-Chávez P, Saavedra-Molina A, López-Meza JE, Ochoa-Zarzosa A, et al. Antioxidant Activity of the Essential Oil and its Major Terpenes of Satureja macrostema (Moc. and Sessé ex Benth.) Briq. Pharmacogn Mag. 2018;13(Suppl 4):S875–80. doi: 10.4103/pm.pm_316_17 29491647 PMC5822514

[42] Ćavar S, Maksimović M, Šolić ME, Jerković-Mujkić A, Bešta R. Chemical composition and antioxidant and antimicrobial activity of two Satureja essential oils. Food Chemistry. 2008;111(3):648–53. doi: 10.1016/j.foodchem.2008.04.033

[43] Obeidnejad E, Kavoosi G, Saharkhiz MJ, Niakousari M. Functional properties and anti‐hyperglycaemia capacity of Satureja essential oil: stabilisation of essential oil in gelatin and physico‐chemical properties characterisation. Int J of Food Sci Tech. 2024;59(4):2570–80. doi: 10.1111/ijfs.16996

[44] Obeidnejad E, Kavoosi G, Saharkhiz MJ. Antioxidant, anti-amylase, anti-lipase, and efficiency of Satureja fatty acid on the anti-inflammatory parameters in lipopolysaccharide-stimulated macrophage through Nrf2/NF-kB/NADH oxidase pathway. Sci Rep. 2024;14(1):12490. doi: 10.1038/s41598-024-63205-6 38821994 PMC11143312

[45] Zarei N, De Craene J-O, Shekarforoush SS, Nazifi S, Golmakani M-T, Giglioli-Guivarc’h N, et al. Anti-obesity potential of selected medicinal plants: A focused study on in vitro inhibitory effects on lipase, α-amylase and α-glucosidase enzymes. J Ethnopharmacol. 2025;348:119733. doi: 10.1016/j.jep.2025.119733 40228587

[46] Askin B, Bayburtluoglu T, Yazici SO. A comparative study on the phenolic extraction of total hydro-alcoholic extract of Satureja hortensis L. for bioactive properties. Kadirli Uygulamalı Bilimler Fakültesi Dergisi. 2025;5(1):29–46.

[47] Pavlović MO, Lunić T, Graovac S, Mandić M, Repac J, Gašić U, et al. Extracts of selected Lamiaceae species as promising antidiabetics: Chemical profiling, in vitro and in silico approach combined with dynamical modeling. Industrial Crops and Products. 2022;186:115200. doi: 10.1016/j.indcrop.2022.115200

[48] Vladimir-Knežević S, Blažeković B, Kindl M, Vladić J, Lower-Nedza AD, Brantner AH. Acetylcholinesterase inhibitory, antioxidant and phytochemical properties of selected medicinal plants of the Lamiaceae family. Molecules. 2014;19(1):767–82. doi: 10.3390/molecules19010767 24413832 PMC6271370

[49] Mihajilov-Krstev T, Radnović D, Kitić D, Jovanović V, Mitić V, Stojanović-Radić Z, et al. Chemical composition, antimicrobial, antioxidative and anticholinesterase activity of Satureja Montana L. ssp montana essential oil. Open Life Sciences. 2014;9(7):668–77. doi: 10.2478/s11535-014-0298-x

